# Evaluating the Cultural Relevance of AI Therapist Responses for Chinese American Caregivers of Older Adults

**DOI:** 10.1093/geroni/igaf122.1744

**Published:** 2025-12-31

**Authors:** Yanjing Liang, Jingyi Li, Serena Jinchen Xie, Shumenghui Zhai, Xuehong Fan, Weichao Yuwen

**Affiliations:** University of Washington, Seattle, Washington, United States; University of Washington Tacoma, Tacoma, Washington, United States; University of Washington, Seattle, Washington, United States; Pacific Lutheran University, Seattle, Washington, United States; University of Washington, Seattle, Washington, United States; University of Washington Tacoma, Tacoma, Washington, United States

## Abstract

Chinese American caregivers underuse professional mental health services despite the high demand for caregiving support. A key barrier is the lack of culturally and linguistically appropriate programs. Large Language Model (LLM)-based AI chatbots offer potential mental health support, but many lack cultural adaptation, limiting effectiveness and acceptance. Advances in Natural Language Processing (NLP) provide opportunities for cost-effective cultural adaptations. This study evaluates the cultural relevance of LLM-generated responses with (WCC) and without (NCC) cultural context. We previously developed a chatbot to support Chinese American caregivers for their self-care. Community partners created culturally rich client inputs, which we used to generate chatbot’s responses. The research team finalized 40 prompts, which were used to generate responses via GPT-4o. Chinese American caregivers evaluated these responses using the Cultural Competency Measure (CCM), Empathy Scale, and Cultural Relevance Questionnaire (CRQ) via Qualtrics. Data from 36 participants (collected until March 11, 2025) were analyzed using descriptive statistics and the Wilcoxon signed-rank test. Results showed that WCC responses had significantly higher CCM, empathy, and CRQ median scores than NCC (p < 0.05). These findings suggest that GPT-4o, when combined with cultural context prompts, generates more culturally appropriate responses. This study highlights the importance of culturally adapted AI-driven interventions in addressing mental health disparities among Chinese American caregivers, offering an accessible solution to enhance caregiver support. By demonstrating the effectiveness of culturally contextualized LLM responses, these findings underscore the potential for integrating AI tools into gerontological nursing to improve caregiver well-being and, ultimately, the quality-caring for older adults.

